# Density, Viscosity and Surface Tension of Binary Mixtures of 1-Butyl-1-Methylpyrrolidinium Tricyanomethanide with Benzothiophene

**DOI:** 10.1007/s10953-014-0257-1

**Published:** 2014-11-14

**Authors:** Urszula Domańska, Marta Królikowska, Klaudia Walczak

**Affiliations:** 1 Department of Physical Chemistry, Faculty of Chemistry, Warsaw University of Technology, Noakowskiego 3, 00-664 Warsaw, Poland; 2 Thermodynamic Research Unit, School of Chemical Engineering, University of KwaZulu-Natal, Howard College Campus, King George V Avenue, Durban, 4001 South Africa

**Keywords:** **(**[BMPYR][TCM] + benzothiophene), Experimental density, Dynamic viscosity, Surface tension, Molecular interactions, Thermodynamics

## Abstract

The effects of temperature and composition on the density and viscosity of pure benzothiophene and ionic liquid (IL), and those of the binary mixtures containing the IL 1-butyl-1-methylpyrrolidynium tricyanomethanide ([BMPYR][TCM] + benzothiophene), are reported at six temperatures (308.15, 318.15, 328.15, 338.15, 348.15 and 358.15) K and ambient pressure. The temperature dependences of the density and viscosity were represented by an empirical second-order polynomial and by the Vogel–Fucher–Tammann equation, respectively. The density and viscosity variations with compositions were described by polynomials. Excess molar volumes and viscosity deviations were calculated and correlated by Redlich–Kister polynomial expansions. The surface tensions of benzothiophene, pure IL and binary mixtures of ([BMPYR][TCM] + benzothiophene) were measured at atmospheric pressure at four temperatures (308.15, 318.15, 328.15 and 338.15) K. The surface tension deviations were calculated and correlated by a Redlich–Kister polynomial expansion. The temperature dependence of the interfacial tension was used to evaluate the surface entropy, the surface enthalpy, the critical temperature, the surface energy and the parachor for pure IL. These measurements have been provided to complete information of the influence of temperature and composition on physicochemical properties for the selected IL, which was chosen as a possible new entrainer in the separation of sulfur compounds from fuels. A qualitative analysis on these quantities in terms of molecular interactions is reported. The obtained results indicate that IL interactions with benzothiophene are strongly dependent on packing effects and hydrogen bonding of this IL with the polar solvent.

**Electronic supplementary material:**

The online version of this article (doi:10.1007/s10953-014-0257-1) contains supplementary material, which is available to authorized users.

## Introduction

New international regulations require the removal of low level sulfur compounds such as thiophene, benzothiophene, methyldibenzothiophenes, 4,6-dibenzothiophenethiols, thioethers, and disulfides from fuels. From an industrial point of view these are new challenges to decrease the sulfur content in diesel fuel in the USA and Europe [1, 2]. The total sulfur content in European gasoline and diesel fuels must be at a maximum concentration of 10 ppm [2]. Thus, the emission of sulfur from petrol and diesel oils, which is linked to acid rain phenomena, plays a crucial role in pollution problems of large conglomerates. The hydrodesulfurization (HDS) process, the established method used in industrial technologies to remove organic sulfur compounds from fuels, cannot achieve these low sulfur targets and uses higher temperature, higher pressure, larger reactor volumes and more active catalysts [3]. Therefore, the easy liquid–liquid equilibrium (LLE) extraction process is proposed for deep desulfurization with ionic liquids (ILs) [4–7]. ILs are not volatile, are nonflammable and show excellent solvation capacity mainly via hydrogen bonding. Great effort has been made to design and synthesize novel ILs to match potential applications such as media for extraction processes [4–14]. ILs that consist of a short alkane chain with polar groups such as oxygen, or nitryle, or hydroxyl substituent in cation and cyano-subgroups in the anion, are expected to be good entrainers in many separation processes. The application of ILs in the desulfurization process has already been reported in the literature [7–14]. It is therefore a challenge to design ILs that incorporate progressively larger extraction selectivity, while maintaining viscosity, density and surface tension convenient for a new technology.

Several recent attempts have focused on the design and synthesis of ILs with high selectivity for the separation of sulfur compounds from alkanes. The 1-alkylpyrrolidinium-based ILs with different anions [14] have been recently studied in our laboratory in ternary LLE (IL + thiophene + heptane) mixtures at *T* = 298.15 K. The highest selectivity (*S*_max_ = 133.4) with high solute distribution ratio (*β* = 3.47) was found for 1-butyl-1-methylpyrrolidinium tricyanomethanide [BMPYR][TCM] [14]. However, larger extraction parameters are presented by 1-ethyl-3-methylimidazolium tricyanomethanide, [EMIM][TCM] [13]. Promising results in ternary LLE measurements were obtained also with 1-ethyl-3-methylimidazolium bis{(trifluoromethyl)sulfonyl}imide, [EMIM][NTf_2_] [11], and 1-butyl-1-methylpyrrolidinium tetracyanoborate, [BMPYR][TCB] [14].

The current work represents a continuation of our systematic study on desulfurization of fuels. We have just reported experimental ternary LLE data for three ILs, which we expected to show high selectivity for the extraction of thiophene: 1-butyl-1-methylpyrrolidinium trifluoromethanesulfonate, [BMPYR][CF_3_SO_3_], 1-butyl-1-methylpyrrolidinium tricyanomethanide, [BMPYR][TCM], and 1-hexyl-3-methylimidazolium tetracyanoborate, [HMIM][TCB] [14]. Therefore, we thought it quite possible that the incorporation of the pyrrolidinium cation and the tricyanomethanide anion would improve the extraction activity of benzothiophene. The ternary systems {IL (1) + thiophene or benzothiophene (2) + heptane (3)} were measured at *T* = 308.15 K and ambient pressure. The [BMPYR][TCM] was found to show high selectivity in the desulfurization process. Recently, very good results for the extraction of sulfur compounds from model mixtures of real fuels were also obtained with tricyanomethanide-based, [TCM]^−^, ILs [7]. The extraction of thiophene and dibenzothiophene (about 95 wt%) was reported for their simultaneous separation from alkanes with pyridine-based and imidazolium-based ILs [7].

Liquid–liquid extraction, such as extraction of benzothiophene from petrol and diesel oils, is greatly affected by viscosity and liquid surface tension. Knowledge about the physicochemical properties such as density, viscosity, or surface tension and thermodynamic surface properties is necessary in order to design any process involving ILs on an industrial scale [15]. The excess functions calculated from viscosity and density of binary systems play a very important role in the understanding of molecular interactions that exist in the bulk of liquids and on the surface. Recently, we published new data for *N*-octylisochinolinium bis{(trifluoromethyl)sulfony}imide, [OiQuin][NTf_2_], for possible extraction of 2-phenylethanol from the aqueous phase [16], and for 1-alkyl-cyanopyridinium-based ILs [17] as well as [EMIM][TCM] [18], for possible extraction of sulfur compounds from fuels. As in all hydrogen-bonded liquids, the structural organization of constituents makes ILs behave as very viscous fluids. The high viscosity of ILs is widely known and usually destroys the mass transport of extractants in new IL-entrainers and limits their generalized use for a variety of applications. Studies of physicochemical properties, besides helping in deciding limits of increase in temperature for the desired applications, are expected to reflect the molecular interactions in binary systems.

Liquid surface tension as an equilibrium thermodynamic property is important for engineering aspects related to use of the IL as an entrainer. Surface tension of a liquid is related to the intermolecular interaction potential energy and the liquid interfacial microstructure. Knowledge of the impact of temperature on the surface tensions of fluids is essential for most industrial applications. In recent years, measurements of the experimental surface tension data has been focused mainly on imidazolium-based ILs, which are air and moisture stable, and their binary solutions with alcohols and water [19, 20]. The surface tensions of ILs are usually lower than that of water (71.98 mN·m^−1^ at *T* = 293.15 K, 0.1 MPa, [21]); for example, for 1-ethyl-3-methylimidazolium tricyanomethanide it is 50.94 mN·m^−1^ at *T* = 298.15 K [18].

The current work represents a continuation of our systematic study on desulfurization of fuels and physicochemical properties of ILs. We report an experimental investigation of the density, viscosity and surface tension for the pure IL, [BMPYR][TCM], and benzothiophene, as well as of binary mixtures containing ([BMPYR][TCM] + benzothiophene) as a function of temperature and composition at ambient pressure.

Using the quasi-linear variation of surface tension with temperature observed for the pure IL, the surface thermodynamic properties, such as surface entropy, surface enthalpy, surface energy, the critical temperature and parachor were determined. The data obtained were analyzed to determine the effect of temperature on fundamental physicochemical and thermodynamic properties.

## Experimental Section

### Materials

The sample of 1-butyl-1-methylpyrrolidinium tricyanomethanide, [BMPYR][TCM], was from Iolitec (≥0.98 mass fraction), *M*_w_ = 232.32 g·mol^−1^, CAS No. 878027-72-6. The sample was dried for several days at 300 K under reduced pressure to remove volatile impurities and trace water and was then stored in a desiccator under an inert atmosphere. Benzothiophene, Sigma Aldrich Chemie GmbH (≥0.99 mass fraction), CAS No. 95-15-8, was stored over freshly activated molecular sieves (4Å, Union Carbide).

**Scheme 1:**
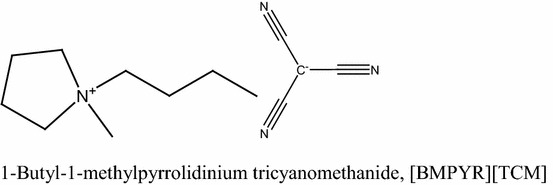
The chemical structure of [BMPYR][TCM]

The structure of [BMPYR][TCM] is shown in Scheme 1. Physical properties: density, *ρ*, dynamic viscosity, *η*, and surface tension, *σ*, of pure IL and benzothiophene, together with the literature data, are listed in Table 1 [22–24].

**Table 1:** Physical properties: density, *ρ*, dynamic viscosity, *η* and surface tension, *σ* of pure ionic liquid and benzothiophene at *T* = 308.15 K

	*ρ* ^expt.^ (g·cm^−3^)	*ρ* ^lit.^ (g·cm^−3^)	*η* ^expt.^ (mPa·s)	*η* ^lit.^ (mPa·s)	*σ* ^expt.^ (mN·m^−1^)	*σ* ^lit.^ (mN·m^−1^)
[BMPYR][TCM]	1.00076	1.00066^a^	20.565	–	48.04	–
Benzothiophene	1.15081	1.15055^b^	2.941	2.517^c^	34.49	42.6^c^
		1.14860^c^ (309.15 K)				

^a^Extrapolated value from Ref. [22]

^b^Ref. [23]

^c^Ref. [24]

The water content was analyzed by Karl Fischer titration (method TitroLine KF). The sample of IL, or solvent, was dissolved in methanol and titrated in steps of 0.0025 cm^3^. The error in the water content is ±10 ppm by mass for the 3 cm^3^ of injected IL. The water content in solvents used was less than 350 ppm by mass.

### Density Measurements

The densities of the chemicals used and their mixtures were measured using an Anton Paar GmbH 4500 vibrating-tube densimeter (Graz, Austria) thermostatted over the (308.15–358.15) K temperature range. The temperature was controlled with two integrated Pt 100 platinum thermometers providing good precision of (±0.01 K). The densimeter includes an automatic correction for the viscosity of the sample. The apparatus is precise to within 1 × 10^−5^ g·cm^−3^, and the overall uncertainty of the measurements was estimated to be better than 5 × 10^−5^ g·cm^−3^. The calibration of the densimeter was performed at atmospheric pressure using doubly distilled and degassed water {CAS: 77-32-18-5; Anton Paar GmbH, liquid density standard, density, 0.99820 ± 0.00002 g·cm^−3^ (293.15 K); literature density 0.9982323 g·cm^−3^ (293.15 K, KNOVEL DIPPR); conductivity, *κ* = 8 μS}, specially-purified benzene (CAS: 71-43-2; standard CHE USC 11; CHEMIPAN, Poland, 0.9999 in mass fraction), and dried air. The data are similar to the literature data of different ILs [14, 16–18].

### Viscosity Measurements

Viscosity measurements were carried out in an Anton Paar BmbH AMVn (Graz, Austria) programmable rheometer, with a nominal uncertainty of ±0.1 % and reproducibility <0.05 % for viscosities from 2.54 to 370 mPa·s. Temperature was controlled internally with a precision of ± 0.01 K in the range from 308.15 to 358.15 K. The diameter of the capillary was 1.8 mm for viscosities from 2.5 to 70 mPa·s. The diameter of the balls was 1.5 mm.

### Surface Tension Measurements

The surface tension measurements were made with a Tensiometer (KSV Sigma 701 System Finland) using a Du-Noüy ring taking into account the Zuidema Waters correction. Measurements were performed using the ring method that is widely used [25] since the studies of Harkins and Jordan [26] that improved the accuracy and established tables of correction factors based on the work of Freund and Freund [27]. The force acting on the balance was recorded with respect to time. The maximum value of the downward force was used to calculate the surface tension. All measurements were repeated three to five times. The equipment has both a control and a mechanic unit that are connected to a PC-controlled instrument for the precise measurement of a liquid with an uncertainty of ±0.04 mN·m^−1^. Temperature was maintained at the desired value within ±0.1 K.

## Results and Discussion

### Effect of Temperature and Composition on Density and Viscosity

The experimental data of density, *ρ*, and dynamic viscosity, *η*, as a function of mole fraction, *x*_1_, of the {[BMPYR][TCM] (1) + benzothiophene (2)} system at different temperatures are listed in Table 2.

**Table 2:** Experimental density, *ρ*, excess molar volume, *V* ^E^, dynamic viscosity, *η*, and viscosity deviation, ∆*η* for the {[BMPYR][TCM] (1) + benzothiophene (2)} binary system as a function of temperature and composition

*X*_1_	308.15	318.15	328.15	338.15	348.15	358.15
*ρ* (g·cm^−3^)						
1.0000	1.00076	0.99469	0.98868	0.98273	0.97684	0.97100
0.8888	1.01123	1.00505	0.99893	0.99287	0.98687	0.98094
0.7788	1.02282	1.01653	1.01030	1.00412	0.99800	0.99193
0.6435	1.03924	1.03277	1.02637	1.02003	1.01374	1.00750
0.4733	1.06393	1.05721	1.05054	1.04392	1.03735	1.03081
0.3889	1.07814	1.07125	1.06440	1.05759	1.05082	1.04410
0.3233	1.09010	1.08302	1.07599	1.06900	1.06205	1.05512
0.0000	1.15081	1.14183	1.13285	1.12388	1.11490	1.10592
*V* ^E^ (cm^3^·mol^−1^)						
1.0000	0.0000	0.0000	0.0000	0.0000	0.0000	0.0000
0.8888	−0.3639	−0.3787	−0.3946	−0.4114	−0.4295	−0.4533
0.7788	−0.7035	−0.7356	−0.7698	−0.8040	−0.8406	−0.8794
0.6435	−1.1136	−1.1626	−1.2170	−1.2746	−1.3344	−1.3977
0.4733	−1.5617	−1.6362	−1.7141	−1.7951	−1.8804	−1.9675
0.3889	−1.7296	−1.8128	−1.8989	−1.9873	−2.0795	−2.1774
0.3233	−1.8067	−1.8904	−1.9789	−2.0704	−2.1664	−2.2641
0.0000	0.0000	0.0000	0.0000	0.0000	0.0000	0.0000
*η* (mPa·s)						
1.0000	20.56	15.44	12.00	9.59	7.81	6.47
0.8888	18.76	14.10	10.97	8.77	7.16	5.93
0.7788	17.03	12.77	9.95	7.96	6.49	5.38
0.6435	14.78	11.13	8.69	6.96	5.67	4.74
0.4733	11.92	9.01	7.10	5.68	4.65	3.88
0.3889	10.45	7.87	6.19	4.98	4.10	3.44
0.3233	9.23	6.89	5.41	4.36	3.59	3.03
0.0000	2.94	2.06	1.74	1.49	1.29	1.14
*∆η* (mPa·s)						
1.0000	0.00	0.00	0.00	0.00	0.00	0.00
0.8888	0.15	0.15	0.11	0.08	0.07	0.05
0.7788	0.36	0.29	0.22	0.15	0.11	0.09
0.6435	0.50	0.46	0.34	0.25	0.18	0.16
0.4733	0.64	0.61	0.50	0.36	0.27	0.22
0.3889	0.66	0.61	0.45	0.34	0.27	0.22
0.3233	0.59	0.50	0.3	0.25	0.19	0.16
0.0000	0.00	0.00	0.00	0.00	0.00	0.00

^a^Standard uncertainties *u* are as follows: *u*(*x*_1_) = ± 1 × 10^−4^, *u*(*ρ*) = ± 1 × 10^−4^ g·cm^−3^, *u*_r_(*η*) = ± 3 % and *u*(*T*) = ± 0.01 K

Fit parameters with *R* ^2^ = 1 for the empirical correlation (see Eqs. 1 and 2) of the density as a function of temperature (*a*_0_, *a*_1_and *a*_2_) and concentration (*b*_*i*_), for pure substances and for mixtures, are listed in Tables 1S and 2S in the supplementary material (SM), respectively:

1 $$ \rho = a_{2} T^{2} + a_{1} T + a_{0} $$

2 $$ \rho = b_{4} x_{1}^{4} + b_{3} x_{1}^{3} + b_{2} x_{1}^{2} + b_{1} x_{1} + b_{0} $$

**Fig. 1:**
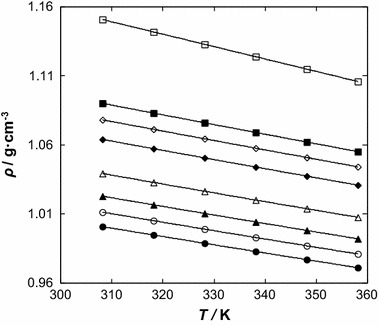
Density, *ρ*, for the {[BMPYR][TCM] (1) + benzothiophene (2)} binary mixtures as a function of temperature at different IL mole fraction, *x*_1_: (*filled circle*) 1.0000, (*open circle*) 0.8888, (*filled triangle*) 0.7788, (*open triangle*) 0.6435, (*filled diamond*) 0.4733, (*open diamond*) 0.3889, (*filled square*) 0.3233, and (*open square*) 0.0000. *Solid lines* represent the polynomial with parameters given in Table 1S in the supplementary material

**Fig. 2:**
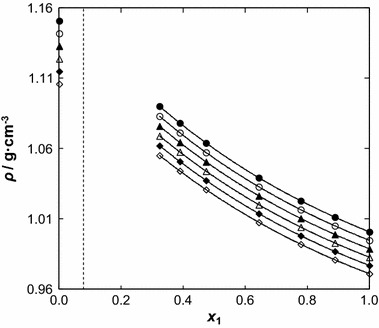
Density, *ρ*, for the {[BMPYR][TCM] (1) + benzothiophene (2)} binary mixtures as a function of concentration *x*_1_ at different temperatures: (*filled circle*) 308.15 K, (*open circle*) 318.15, (*filled triangle*) 328.15 K, (*open triangle*) 338.15 K, (*filled diamond*) 348.15 K, and (*open diamond*) 358.15 K. *Solid lines* represent the polynomial with parameters given in Table 2S in the supplementary material. The *dotted line* represents the immiscibility gap [14]

The density of [BMPYR][TCM] is lower than that of benzothiophene, but the viscosity is almost ten times higher. The densities of the IL range in values from 1.00076 g·cm^−3^ at *T* = 308.15 K (*ρ* = 1.00066 g·cm^−3^, extrapolated value from [22]) to 0.97100 g·cm^−3^ at *T* = 358.15 K, and of benzothiophene from 1.15081 g·cm^−3^ at *T* = 308.15 K to 1.10592 g·cm^−3^ at *T* = 358.15 K. Immiscibility in the binary solutions of {[BMPYR][TCM] (1) + benzothiophene (2)} was observed in our ternary LLE measurements [13, 28]. The data presented in this work do not cover the compositions at the immiscibility gap (see Figs. 1, 2).

The viscosity decreases with increasing benzothiophene content. The dynamic viscosity of the pure IL and the mixtures as a function of temperature, through the whole composition range, was correlated by the well-known Vogel–Fulcher–Tammann, VFT equation [29–31],

3 $$ \eta = CT^{0.5} \exp \left(\frac{D}{{T - T_{0} }}\right) $$

**Fig. 3:**
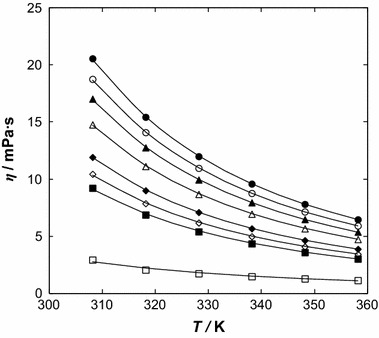
Dynamic viscosity, *η*, as a function of temperature for the {[BMPYR][TCM] (1) + benzothiophene (2)} binary mixtures at different IL mole fraction, *x*_1_: (*filled circle*) 1.0000, (*open circle*) 0.8888, (*filled triangle*) 0.7788, (*open triangle*) 0.6435, (*filled diamonds*) 0.4733, (*open diamonds*) 0.3889, (*filled square*) 0.3233 and (*open square*) 0.0000. *Solid lines* represent the VFT equation with parameters given in Table 3; the *dotted line* represents the immiscibility gap [14]

The fit parameters, determined empirically, are in general *C,* *D* and *T*_0_ when a linear relation is observed between logarithmic value of *ηT* ^0.5^ and (*T* − *T*_0_)^−1^. For the best correlation of the experimental curves, the value of *T*_0_ = 118.01 K (*T*_g,1_ = 178.01 K [32] −60 K) was used in the calculations. A single value of the parameter *T*_0_ was used for different concentrations. Figure 3 depicts the dynamic viscosity as a function of temperature. The temperature dependence of viscosity becomes distinctly nonlinear, especially at low benzothiophene content. The parameters *C* and *D* from Eq. 3 change smoothly with composition for the system, as shown in Table 3S in the supplementary material.

The composition dependence of viscosity was described by the following polynomial:

4 $$ \eta = c_{3} x_{1}^{3} + c_{2} x_{1}^{2} + c_{1} x_{1}^{{}} + c_{0} $$

**Fig. 4:**
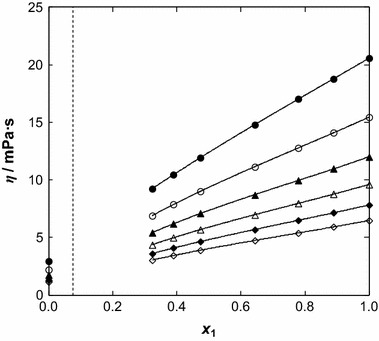
Dynamic viscosity, *η*, as a function of ionic liquid mole fraction, *x*_1_, for the {[BMPYR][TCM] (1) + benzothiophene (2)} binary mixtures at different temperatures: (*filled circle*) 308.15 K, (*open circle*) 318.15, (*filled triangle*) 328.15 K, (*open triangle*) 338.15 K, (*filled diamond*) 348.15 K, and (*open diamond*) 358.15 K. *Solid lines* represent the polynomial with parameters given in Table 4S in the supplementary material; the *dotted line* represents the immiscibility gap [14]

The parameters of the correlation are listed in Table 4S in the supplementary material and the calculated lines are shown in Fig. 4. The dynamic viscosity of the IL changes from 20.56 mPa·s at *T* = 308.15 K to 6.47 mPa·s at *T* = 358.15 K, and for benzothiophene from 2.94 mPa·s at *T* = 308.15 K to 1.14 mPa·s at *T* = 358.15 K. The values of viscosity presented in this work are higher than that reported for [EMIM][TCM] [18], which was suggested as a very good entrainer for the extraction of sulfur compounds from alkanes. ILs exhibit high viscosities that are usually higher than those for molecular organic solvents. Both density and viscosity decrease with an increase of temperature.

The values of excess molar volumes, *V*_*m*_^E^, of the mixtures formed from two polar compounds are the result of a number of effects which may contribute terms differing in sign. Disruption of H-bonds in the IL molecules makes a positive contribution, but specific interaction between two dissimilar molecules makes negative contributions to *V*_*m*_^E^. The free-volume effect, which depends on differences in the characteristic pressures and temperatures of the components (described by Flory formalism [33]), makes a negative contribution. Packing effects or conformational changes of the molecules in the mixtures are more difficult to categorize. However, interstitial accommodation and the effect of the condensation give further negative contributions.

**Fig. 5:**
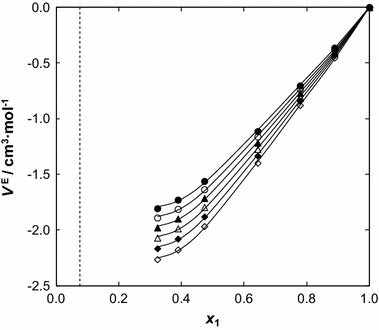
Excess molar volume, *V* ^E^, versus the ionic liquid mole fraction, *x*_1_, for the {[BMPYR][TCM] (1) + benzothiophene (2)} binary mixtures at different temperatures: (*filled circle*) 308.15 K, (*open circle*) 318.15, (*filled triangle*) 328.15 K, (*open triangle*) 338.15 K, (*filled diamond*) 348.15 K, and (*open diamond*) 358.15 K. The *solid line* represented the Redlich–Kister equation with parameters given in Table 5S; the *dotted line* represents the immiscibility gap [14]

Experimental excess molar volume *V*_*m*_^E^ data of {[BMPYR][TCM] (1) + benzothiophene (2)} are listed in Table 2. The data were correlated by the well-known polynomial Redlich–Kister equation (Eq. 5):

5 $$ V_{m}^{\text {E}} = x_{1} (x_{1} - 1)\sum\limits_{i = 0}^{i = 3} {A_{i} (1 - 2x_{1} )}^{i - 1} $$

6 $$ \sigma_{V} = \left[ \left\{ \sum\limits_{i = 1}^{n} {(V_{m}^{{\text {E}({\text{exp}}.)}} - V_{m}^{{\text {E} ( {\text{calc}} . )}} )} /(n - k) \right\} \right]^{1/2} $$

where *x*_1_ is the mole fraction of the IL and *V*_*m*_^E^ is the molar excess volume. The values of the parameters (*A*_*i*_) were determined using the least-squares method. The fit parameters are summarized in Table 5S in the supplementary material, along with the corresponding standard deviations, *σ*_*V*_, for the correlations (Eq. 6), where *n* is the number of experimental points and *k* is the number of coefficients. The values of *V*_*m*_^E^, as well as the Redlich–Kister fits, are plotted in Fig. 5 as a function of the mole fraction. The *V*_*m*_^E^ values exhibit negative deviations from ideality over the entire composition range. The graph also shows the unsymmetrical variation of these excess molar volumes with composition. The minimum of *V*_*m*_^E^ is close to −1.8067 cm^3^·mol^−1^, at mole fraction *x*_1_ = 0.3233 (at *T* = 308.15 K) and is shifted to lower values of mole fraction of the IL. The values of *V*_*m*_^E^ decrease as the temperature increases. The strength of interactions between the IL and benzothiophene is at its highest and most negative at the higher temperature. This has to be the result of a more efficient packing effect rather than due to interactions at higher temperature.

The values of the excess dynamic viscosity, Δ*η*, are listed in Table 2. These values were correlated with the following Redlich–Kister equation:

7 $$ \varDelta \eta = x_{1} (x_{1} - 1)\sum\limits_{i = 0}^{i = 3} {B_{i} (1 - 2x_{1} )}^{i - 1} $$

8 $$ \sigma_{\Delta \eta } \, = \,\left[ { \left\{ \sum\limits_{{{{i}} = 1}}^{{n}} {(\Delta \eta^{{{ \exp } }} - \Delta\eta^{{{\text{calc}} }} )}  /({{n}} - {{k}}) \right\} } \right]^{1/2} $$

**Fig. 6:**
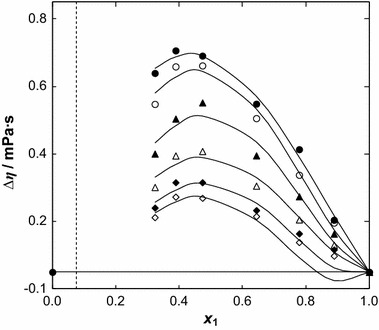
Dynamic viscosity deviation, Δ*η*, versus ionic liquid mole fraction, *x*_1_ for the {[BMPYR][TCM] (1) + benzothiophene (2)} binary mixtures at different temperatures: (*filled circle*) 308.15 K, (*open circle*) 318.15, (*filled triangle*) 328.15 K, (*open triangle*) 338.15 K, (*filled diamond*) 348.15 K and (*open diamond*) 358.15 K. The *solid line* represents the Redlich–Kister equation with parameters given in Table 6S in the supplementary material; the *dotted line* represents the immiscibility gap [14]

The parameters are listed in Table 6S in the supplementary material. Figure 6 shows the positive values of the excess dynamic viscosity for this binary system with Δ*η*_max_ minimally shifted to a lower IL mole fraction.

### Effect of Temperature and Composition on the Surface Tension

The values of surface tension, *σ*, of [BMPYR][TCM] at different temperatures (308.15 K to 338.15 K) are listed in Table 3. Within the present study, the surface tension of [BMPYR][TCM] at *T* = 308.15 K is 48.04 mN·m^−1^. This value is much higher than those for other, mainly imidazolium, ILs [19], but is very similar to the tricyanamide-based IL [EMIM][TCM] measured by us (49.91 mN·m^−1^ at *T* = 298.15 K) [18]. The surface tension is much higher for the IL than for benzothiophene and decreases with increasing concentration of benzothiophene, implying that the benzothiophene molecules tend to adsorb at the air–solution interface due to it hydrophobicity. The surface tension decreases with an increase of temperature, which is typical for organic solvents.

**Table 3:** Experimental surface tension, *σ*, and surface tension deviation, *∆σ*, for the {[BMPYR][TCM] (1) + benzothiophene (2)} binary system as a function of temperature and composition

*x*_1_	308.15	318.15	328.15	338.15
*σ* (mN·m^−1^)				
1.0000	48.04	47.59	47.04	46.39
0.8888	47.63	47.08	46.59	45.99
0.6435	46.55	46.08	45.41	44.84
0.4733	45.65	45.05	44.39	43.60
0.3889	45.03	44.37	43.61	42.82
0.3233	44.24	43.58	42.80	42.10
0.0000	34.49	33.40	32.12	30.41
*Δσ* (mN·m^−1^)				
1.0000	0.00	0.00	0.00	0.00
0.8888	1.10	1.07	1.20	1.37
0.6435	3.34	3.55	3.69	4.15
0.4733	4.75	4.93	5.21	5.63
0.3889	5.27	5.45	5.69	6.20
0.3233	5.36	5.59	5.86	6.52
0.0000	0.00	0.00	0.00	0.00

^a^Standard uncertainties *u* are as follows: *u*(*x*_1_) = ± 1 × 10^−4,^ *u*(*σ*) = ± 0.1 mN·m^−1^ and *u*(*T*) = ± 0.01 K

The correlation of the surface tension as a function of temperature and composition was represented with the equations:

9 $$ \sigma = d_{1} T + d_{0} $$

10 $$ \sigma = e_{3} x_{1}^{3} + e_{2} x_{1}^{2} + e_{1} x_{1} + e_{0} $$

**Fig. 7:**
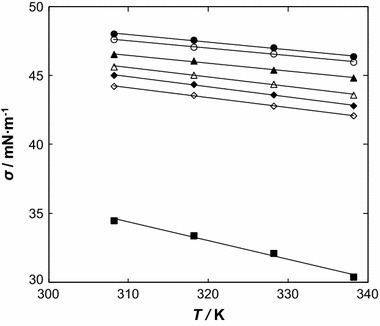
Surface tension, *σ*, as a function of temperature for the {[BMPYR][TCM] (1) + benzothiophene (2)} binary mixtures at different IL mole fraction, *x*_1_: (*Black filled circle*) 1.0000, (*open circle*) 0.8888, (*filled triangle*) 0.7788, (*open triangle*) 0.6435, (*filled diamond*) 0.4733, (*open diamond*) 0.3889, (*filled square*) 0.3233, and (open square) 0.0000. *Solid lines* represent the polynomial with parameters given in Table 7S in the supplementary material

**Fig. 8:**
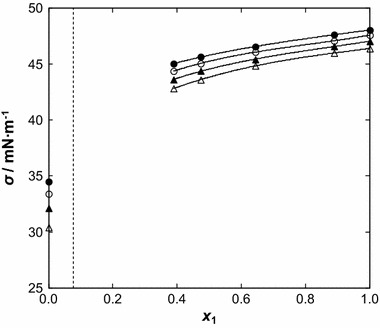
Surface tension, *σ*, as a function of IL mole fraction, *x*_1_, for the {[BMPYR][TCM] (1) + benzothiophene (2)} binary mixtures at different temperatures: (*filled circle*) 308.15 K, (*open circle*) 318.15, (*filled triangle*) 328.15 K, and (*open triangle*) 338.15 K. *Solid lines* represent the polynomial with parameters given in Table 8S in the supplementary material

The obtained parameters are shown in Tables 7S and 8S in the supplementary material for temperature and composition dependences, respectively. The surface tension decreases with an increase of temperature and of benzothiophene content in the binary mixtures (see Figs. 7, 8).

The absence of a breakpoint in this mixture confirms the special interactions observed in the LLE in its ternary system [11–14]. These properties cannot be deduced using phase equilibrium data only. A regularly increasing value of the solution surface tension indicates that the two compounds, the IL and benzothiophene, are present at the gas/liquid interface. The [BMPYR][TCM] IL is a complex molecule, in which the Columbic forces, hydrogen bonds and van der Waals forces all are present in the interaction between the cation and anion, as well as between the dissimilar molecules in the solution, with the hydrogen bonds being probably the most important forces in the IL at higher mole fractions. This can be explained by the high capacity of benzothiophene to form π-π interactions, making possible an easy accommodation of benzothiophene into the IL’s structure. On the other hand benzothiophene, in comparison with alcohols, is not a substance forming associates between similar molecules, and thus theoretically fewer molecules are free to interact with the IL in the solution and adsorb on the air–liquid surface. The surface tension of the {[BMPYR][TCM] + benzothiophene} solutions present formally similar patterns to those measured earlier [EMIM][TCM] [18]. According to our results, the regular decrease of the surface tension observed with decreasing IL mole fraction confirms that this behavior can be explained by strong interaction (IL + benzothiophene) within the investigated mole fraction region (see Fig. 8).

For the better understanding the results of this work, the surface tension deviation (Δ*σ*) was calculated according to the equation:

11 $$ \Delta \sigma = \sigma - \sum\limits_{i = 0}^{2} {x_{i} \sigma_{i} } $$

where *x*_*i*_ and Δ*σ*_*i*_ are the mole fraction and surface tension deviation of component *i*, respectively. The surface tension deviations were correlated by means of the Redlich–Kister equation in the following form:

12 $$ \Delta \sigma = x_{1} (x_{1} - 1)\sum\limits_{i = 0}^{i = 3} {C_{i} (1 - 2x_{1} )}^{i - 1} $$

where *x*_*i*_ and Δ*σ*_*i*_ are the mole fraction and surface tension deviation of component *i*, respectively. The surface tension deviations at different temperatures are listed in Table 3. The values of parameters *C*_*i*_/(mN·m^−1^) have been determined using the least-squares method:

13 $$ \sigma_{\Delta \sigma } = \left[ \left\{ \sum\limits_{i = 1}^{n} {(\Delta \sigma^{{{\text{exp}}}} - \Delta \sigma^{{{\text{calc}}}} )}  /(n - k) \right\} \right]^{1/2} $$

**Fig. 9:**
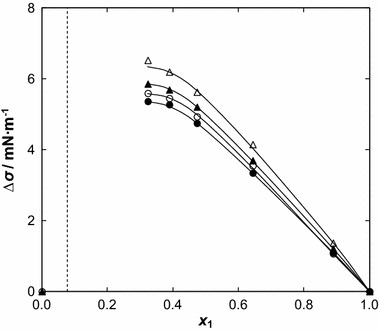
Surface tension deviation, Δ*σ*, versus ionic liquid mole fraction, *x*_1_, for the {[BMPYR][TCM] (1) + benzothiophene (2)} binary mixtures at different temperatures: (*filled circle*) 308.15 K, (*open circle*) 318,15, (*filled triangle*) 328.15 K, and (*open triangle*) 338.15 K. The *solid line* represents the Redlich–Kister equation with parameters given in Table 9S in the supplementary material

The standard deviation, *σ*_Δ*σ*_, is given by the formula (Eq. 13) where *n* is the number of experimental points and *k* is the number of coefficients. The parameters and standard deviations *σ*_Δ*σ*_ are listed in Table 9S in the supplementary material. The values of Δ*σ*_*i*_ are positive for all compositions of {[BMPYR][TCM] (1) + benzothiophene (2^2^)} over the measured composition range as can be seen in Fig. 9. The maximum value of Δ*σ*_*i*_ is 5.36 N·m^−1^ and shifts to a lower mole fraction of the IL, *x*_1_ = 0.3233 at *T* = 388.15 K. Values of Δ*σ*_*i*_ increase with an increase of temperature. This is similar to observations for [EMIM][TCM] [18], but opposite to that observed for (IL + an alcohol) binary mixtures [34–36]. Changes with temperature may be attributed to diminishing of the hydrogen bonding between cation and anion in the IL, and then a new distribution of interactions exists at the surface and in the bulk region.

The measurements of the surface tension as a function of temperature provide the possibility of calculating the surface thermodynamic functions in the measured temperature range (308.15–338.15) K. The surface entropy (*S* ^*σ*^) and the surface enthalpy (*H* ^*σ*^) were calculated from the following equations [37, 38]:

14 $$ S^{\sigma } = - \frac{\text{d}\sigma }{\text{d}T} $$

15 $$ H^{\sigma } = \sigma - T\left( {\frac{\text{d}\sigma }{\text{d}T}} \right) $$

The thermodynamic functions for [BMPYR][TCM] at *T* = 308.15 K are listed in Table 4. The surface entropy is quite high {*S* ^*σ*^ = (55.00 ± 0.05) × 10^−6^ N·m^−1^·K^−1^ at *T* = 308.15 K}, but lower than that for [EMIM][TCM] {*S* ^*σ*^ = (10.61 ± 0.08) × 10^−5^ N·m^−1^·K^−1^ at *T* = 298.15 K [18] }, and higher than those for many ionic liquids [16, 17, 19, 36]. The lower is the surface entropy, the lower is the surface organization of the solution. The lower value of entropy of the IL shows that the partial molar entropy of the IL decreases at the contact between the IL and air in the surface region. The surface enthalpy {*H* ^*σ*^ = (64.98 ± 0.05) × 10^−3^ N·m^−1^ at *T* = 308.15 K} is lower than those observed for [EMIM][TCM] (*T* = 298.15 K) [18] and other ionic liquids [16, 17, 19, 36].

**Table 4:** Surface thermodynamic functions for the pure ionic liquid [BMPYR][TCM] at temperature *T* = 308.15 K: surface entropy, *S* ^σ^, surface enthalpy, *H* ^σ^, critical temperatures, $T_{\text{c}}^{\text{E}}$ and $T_{\text{c}}^{\text{G}}$, and surface energy, *E* ^σ^

10^6^ *S* ^σ^ (N·m^−1^·K^−1^)	10^3^ *H* ^σ^ (N·m^−1^)	$T_{\text{c}}^{\text{E}}$ (K)	$T_{\text{c}}^{\text{G}}$ (K)	*E* ^σ^ (mN·m^−1^)
55.0	64.98	1646	1377	65.46

Because of the negligible vapour pressure of the IL, the critical temperature, (*T*_c_) can be estimated from the measurements of surface tension as a function of temperature according to following two formulae:

16 $$ \sigma \left( {\frac{M}{\rho }} \right)^{{{\raise0.7ex\hbox{$2$} \!\mathord{\left/ {\vphantom {2 3}}\right.\kern-0pt} \!\lower0.7ex\hbox{$3$}}}} = K\left( {T_{\text{c}}^{\text{E}} - T} \right) $$

17 $$ \sigma = E^{\sigma } \left( {1 - \frac{T}{{T_{c}^{\text{G}} }}} \right)^{{{\raise0.7ex\hbox{${11}$} \!\mathord{\left/ {\vphantom {{11} 9}}\right.\kern-0pt} \!\lower0.7ex\hbox{$9$}}}} $$

The critical temperature may be calculated from the Eötvös equation, (Eq. 16) [39], where *K* is a constant, *ρ*/(g·cm^−3^) is the density, *M*/(g·mol^−1^) is the molar mass, *T*/(K) is the temperature of the measured surface tension *σ*/(N·m^−1^), and $T_{\text{c}}^{\text{E}}$ /(K) is the Eötvös critical temperature. The critical temperature can be also calculated from the alternative van der Waals–Guggenheim equation (Eq. 17) for traditional organic liquids [38, 40], where *E* ^*σ*^ is the total surface energy of the IL, which equals the surface enthalpy as long as there is negligible volume change due to thermal expansion at temperatures well removed from the Guggenheim critical temperature $T_{\text{c}}^{\text{G}}$ /(K). The critical temperatures in this work, calculated from (Eqs. 16 and 17) and the total surface energy of the IL, are presented in Table 4. The two obtained values of the critical temperatures differ slightly from each other, ($T_{\text{c}}^{\text{E}}$ /(K) = 1646 and $T_{\text{c}}^{\text{G}}$ /(K) = 1377), and are higher than those of other ILs [16, 17, 19, 36]. The total surface energy of the IL is equal to 65.46 ± 0.05 mN·m^−1^ at *T* = 308.15 K, which is twice as large as that for 1-butyl-3-cyanopyridinium bis{(trifluoromethyl)sulfonyl}imide, [BCN^3^Py][NTf_2_] [17], and similar to [EMIM][TCM] (*T* = 298.15 K) [18]. According to the corresponding states correlations, in both equations (Eqs. 16 and 17) the surface tension becomes null at the critical temperature [40].

Using the definition of parachor (Eq. 18) and the measured density in a range of temperature (308.15 to 338.15) K, the parachor was calculated and the values are listed in Table 5.

**Table 5:** The parachor, *P,* for the pure IL [BMPYR][TCM] in the temperature range *T* = (308.15–338.15) K

*T* (K)	*P* (mN^1/4^·m^−1/4^·cm^3^·mol^−1^)
308.15	616.2 ± 0.1
318.15	618.5 ± 0.1
328.15	620.4 ± 0.1
338.15	622.0 ± 0.1

18 $$ P = \frac{{M\sigma^{1/4}}}{\rho } $$

The obtained value, 616.18 at *T* = 308.15 K (mN·m^−1^)^1/4^·cm^3^·mol^−1^, is similar to many values published earlier for other ILs [16, 17, 19, 36].

## Conclusions

The density, viscosity and surface tension of 1-butyl-1-methylpyrrolidinium tricyanomethanide, [BMPYR][TCM], were measured. The consequences of adding different amounts of benzothiophene and increasing the temperature were investigated. Through density, viscosity and surface tension measurements, it is established that both the increase in temperature and addition of benzothiophene lead to decreases in Coulombic, hydrogen bonding and van der Waals interactions and hence to structural disorder in the ionic liquid.

Negative deviations in the range of measured mole fraction were observed for the excess molar volumes, *V*_*m*_^E^, and positive deviations were observed for both the excess dynamic viscosity, Δ*η*, and surface tension deviation, ∆*σ*. The results show that addition of benzothiophene increases the density but decreases the viscosity and surface tension of the mixture, which results in a loss of structural order at the interface and in the bulk of the IL. The molecular interpretation of the possible interactions for similar and dissimilar molecules, together with the packing effects, were discussed for the measured properties presented here.

The [BMPYR][TCM] IL presents surface tension value of the same order as observed for conventional ionic liquids and much higher than those reported for common organic solvents. The thermodynamic functions of the surface, such as surface entropy and enthalpy, were found to be similar to, or lower than those reported for other ILs. The molecular interpretation of the possible cross-hydrogen bonding between the IL and benzothiophene is difficult because of the immiscibility gap at low IL mole fractions. Thus, the negative excess molar volume data may be interpreted in terms of the packing effect. The packing effects or conformational changes of the molecules play a decisive role in the formation of associates in the solution and of the gas/liquid interface. Values of the parachor derived from the temperature dependence of the surface tension values are believed to be accurate enough for engineering calculations. The comparison made with [EMIM][TCM] [18], measured earlier, shows that the nature of the anion is the dominant factor in determining the extraction process, while changing the cation plays a minor role. Both ILs are similar in their nonaggregation behavior with thiophene or benzothiophene and their surface thermodynamic functions.

The results of the correlations with the second order polynomials, Redlich–Kister equation, and VFT equation for density, viscosity, excess molar volumes, viscosity deviation, surface tension and the surface tension deviation were presented, each with very low standard deviations.

## Electronic supplementary material

Below is the link to the electronic supplementary material.

Supplementary material 1 (DOC 374 kb)
