# Supplementary material for: Density, Viscosity and Surface Tension of Binary Mixtures of 1-Butyl-1-Methylpyrrolidinium Tricyanomethanide with Benzothiophene
Source: J Solution Chem. 2014 Nov 14;43(11):1929–46. doi: 10.1007/s10953-014-0257-1 (PMC4249917; doi:10.1007/s10953-014-0257-1)
Supplement: Supplementary file 1 — Supplementary material 1 (DOC 374 kb) [file 10953_2014_257_MOESM1_ESM.doc]

**Supplementary Material for**

**Density, Viscosity and Surface Tension of Binary Mixtures of 1-Butyl-1-methylpyrrolidinium Tricyanomethanide with Benzothiophene**

**Urszula Domańska · Marta Królikowska · Klaudia Walczak**

**Table 1S** Fit parameters for the empirical density correlation for the {[BMPYR][TCM] (1) + benzothiophene (2)} binary system^a^

| *x*_1_ | 10^7^$a_{2}$ /(g⋅cm^–3^⋅K^–2^) | 10^3^$a_{1}$/ (g⋅cm^–3^⋅K^–1^) | | $a_{0}$/ (g⋅cm^–3^) |
| --- | --- | --- | --- | --- |
| 1.0000 | 2.911 | –7.891 | 1.216 | |
| 0.8888 | 3.089 | –8.117 | 1.232 | |
| 0.7788 | 2.770 | –8.010 | 1.244 | |
| 0.6435 | 2.839 | –8.284 | 1.266 | |
| 0.4733 | 2.321 | –8.170 | 1.311 | |
| 0.3889 | 2.089 | –8.201 | 1.311 | |
| 0.3233 | 1.911 | –8.267 | 1.327 | |
| 0.0000 | 3.471 × 10^–11^ | –8.977 | 1.427 | |

^a^ Parameters of the equation $\rho=a_{2}T^{2}+a_{1}T+a_{0}$

**Table 2S** Coefficients of the polynomial for the correlation of the density as a function of concentration at different temperatures for the {[BMPYR][TCM] (1) + benzothiophene (2)} binary system^a^

| *T* / (K) | *b*_3_ /(g⋅cm^–3^) | *b*_2_ / (g⋅cm^–3^) | *b*_1_ / (g⋅cm^–3^) | *b*_0_ / (g⋅cm^–3^) |
| --- | --- | --- | --- | --- |
| 308.15 | 0.018 | 0.036 | –0.203 | 1.151 |
| 318.15 | 0.022 | 0.026 | –0.195 | 1.145 |
| 328.15 | 0.027 | 0.015 | –0.186 | 1.133 |
| 338.15 | 0.031 | 0.005 | –0.172 | 1.124 |
| 348.15 | 0.036 | –0.005 | –0.168 | 1.115 |
| 358.15 | 0.040 | –0.016 | –0.159 | 1.106 |

^a^ Parameters of the equation $\rho=b_{4}x_{1}^{4}+b_{3}x_{1}^{3}+b_{2}x_{1}^{2}+b_{1}x_{1}+b_{0}$

**Table 3S** Fit parameters of the VFT equation, *C* and *D* for the correlation of dynamic viscosities as a function of temperature for the {[BMPYR][TCM] (1) + benzothiophene (2)} binary system ^a, b^

| *x*_1_ | 10^3^ *C* / (K) | 10^-3^ *D* / (mPa⋅s⋅K^–1^) |
| --- | --- | --- |
| 1.0000 | 1.122 | 3.195 |
| 0.8888 | 1.118 | 2.973 |
| 0.7788 | 1.118 | 2.699 |
| 0.6435 | 1.107 | 2.483 |
| 0.4733 | 1.091 | 2.175 |
| 0.3889 | 1.081 | 1.999 |
| 0.3233 | 1.083 | 1.746 |
| 0.0000 | 0.896 | 1.399 |

a $\eta=CT^{0.5}\exp(\frac{D}{T-T_{0}})$

^b^ *T*_0_ = 118.01 K (*T*_g_ = 178.01 K) from ref. [32]

**Table 4S** Coefficients of the polynomial for the correlation of the dynamic viscosity as a function of concentration at different temperatures for the {[BMPYR][TCM] (1) + benzothiophene (2)} binary system ^a^

| *T* / (K) | *c*_3_ /(mPa⋅s) | *c*_2_ / (mPa⋅s) | *c*_1_ / (mPa⋅s) | *c*_0_ / (mPa⋅s) |
| --- | --- | --- | --- | --- |
| 308.15 | 2.676 | –7.049 | 22.243 | 2.693 |
| 318.15 | 4.868 | –11.353 | 20.700 | 1.232 |
| 328.15 | 5.036 | –1.514 | 17.776 | 0.709 |
| 338.15 | 3.905 | –8.838 | 13.845 | 0.689 |
| 348.15 | 2.752 | –6.209 | 10.515 | 0.764 |
| 358.15 | 2.535 | –5.670 | 8.966 | 0.646 |

^a^ Parameters from the equation $\eta=c_{3}x_{1}^{3}+c_{2}x_{1}^{2}+c_{1}x_{1}+c_{0}$

**Table 5S** Coefficients of the Redlich–Kister equation^a^ for the correlation of the excess molar volume, *V*^E^, for the {[BMPYR][TCM] (1) + benzothiophene (2)} binary system, along with the corresponding standard deviations, $\sigma_{V}$^b^

| *T* / (K) | $A_{1}$/ (cm^3^⋅mol^–1^) | $A_{2}$ / (cm^3^⋅mol^–1^) | $A_{3}$/ (cm^3^⋅mol^–1^) | $\sigma_{V}$**/** (cm^3^⋅mol^–1^) |
| --- | --- | --- | --- | --- |
| 308.15 | –6.0404 | 5.1224 | –2.7639 | 0.0173 |
| 318.15 | –6.3155 | 5.3791 | –2.9909 | 0.0139 |
| 328.15 | –6.6162 | 5.6315 | –3.0991 | 0.0145 |
| 338.15 | –6.9292 | 5.8871 | –3.1944 | 0.0151 |
| 348.15 | –7.2563 | 6.1549 | –3.3052 | 0.0156 |
| 358.15 | –7.5933 | 6.4240 | –3.4637 | 0.0148 |

a $V_{m}^{\text{E}}=x_{1}(x_{1}-1)\sum_{i=0}^{i=3} A_{i}(1-x_{1})^{i-1}$

b $\sigma_{V}=[\{\sum_{i=1}^{n} (V_{m}^{E(\text{expt}.)}-V_{m}^{E\text{(calc.)}})\}/(n-k)\}]^{1/2}$

**Table 6S** Coefficients of the Redlich–Kister equation for the correlation of the dynamic viscosity deviation, *Δη*, for the {[BMPYR][TCM] (1) + benzothiophene (2)} binary system, along with the corresponding standard deviations, *σ*_Δ_*_η_*

| *T* / (K) | *B*_1_/ (mPa⋅s) | *B*_2_/ (mPa⋅s) | *B*_3_/ (mPa⋅s) | $\sigma$/ (mPa⋅s) |
| --- | --- | --- | --- | --- |
| 308.15 | 2.5225 | –0.7889 | –0.3662 | 0.0246 |
| 318.15 | 2.3560 | –0.5725 | –1.0761 | 0.0343 |
| 328.15 | 1.8500 | –0.2017 | –1.3480 | 0.0421 |
| 338.15 | 1.3539 | –0.1704 | –1.0380 | 0.0317 |
| 348.15 | 1.0484 | –0.1735 | –1.2877 | 0.0239 |
| 358.15 | 0.8789 | –0.3029 | – 1.4839 | 0.0294 |

^a^ $\Delta\eta=x_{1}(x_{1}-1)\sum_{i=0}^{i=3} B_{i}(1-x_{1})^{i-1}$; $\sigma_{\Delta\eta}=[\{\sum_{i=1}^{n} (\Delta\eta^{\text{expt}.}-\Delta\eta^{\text{calc}.})\}/(n-k)\}]^{1/2}$

**Table 7S** Fit parameters for the empirical surface tension correlation of the {[BMPYR][TCM] (1) + benzothiophene (2)} binary system a

| *T* / (K) | *d*_1_ **/** (mN⋅m^–1^) | *d*_0_**/** (mN⋅m^–1^) |
| --- | --- | --- |
| 1.0000 | –0.055 | 64.969 |
| 0.8888 | –0.054 | 64.315 |
| 0.6435 | –0.058 | 64.435 |
| 0.4733 | –0.068 | 66.670 |
| 0.3889 | –0.074 | 67.866 |
| 0.3233 | –0.072 | 66.408 |
| 0.0000 | –0.135 | 76.375 |
| 1.0000 | –0.055 | 64.969 |

^a^ Parameters from the equation $\sigma=d_{1}T+d_{0}$

**Table 8S** Coefficients of the polynomial for the correlation of the surface tension as a function of concentration at different temperatures for the {[BMPYR][TCM] (1) + benzothiophene (2)} binary system ^a^

| *T* / (K) | *e*_3_ /(mN⋅m^–1^) | *e*_2_ / (mN⋅m^–1^) | *e*_1_ / (mN⋅m^–1^) | *e*_0_ / (mN⋅m^–1^) |
| --- | --- | --- | --- | --- |
| 308.15 | 3.901 | –10.993 | 14.165 | 40.970 |
| 318.15 | 10.069 | –24.464 | 23.739 | 38.239 |
| 328.15 | 7.268 | –19.092 | 20.911 | 37.960 |
| 338.15 | 5.436 | –16.888 | 20.936 | 36.907 |

^a^ Parameters from the equation $\sigma=e_{3}x_{1}^{3}+e_{2}x_{1}^{2}+e_{1}x_{1}+e_{0}$

**Table 9S** Coefficients of the Redlich–Kister equation for the correlationa of the surface tension deviation, Δ*σ*, for the {[BMPYR][TCM] (1) + benzothiophene (2)} binary system, along with the correspondind standard deviations, $\sigma_{\Delta\sigma}$b

| *T* / (K) | *C*_1_ **/** (mN⋅m^–1^) | *C*_2_**/** (mN⋅m^–1^) | *C*_3_**/** (mN⋅m^–1^) | $\text{σ}_{\Delta\sigma}$**/** (mN⋅m^–1^) |
| --- | --- | --- | --- | --- |
| 308.15 | 18.269 | –15.033 | 7.490 | 0.089 |
| 318.15 | 19.162 | –15.677 | 6.308 | 0.127 |
| 328.15 | 20.004 | –16.269 | 8.073 | 0.048 |
| 338.15 | 21.820 | –17.130 | 8.987 | 0.339 |

^a^$\Delta\sigma=x_{1}\left(x_{1}-1\right)\sum_{i=0}^{i=3} C_{i}(1-2x_{1})^{i-1}$

^b^$\sigma_{\Delta\sigma}\text{=}\left\{\text{\{}\sum_{\text{i}=1}^{\text{n}} (\Delta\sigma^{\exp\text{t.}}-\Delta\sigma^{\text{calc.}})\right\}\text{/}\left(n-k\right)\text{\}}^{\text{1/2}}$
